# Time-Resolved Raman Spectroscopy of Polaron Formation in a Polymer Photocatalyst

**DOI:** 10.1021/acs.jpclett.1c03073

**Published:** 2021-11-03

**Authors:** Verity L. Piercy, Khezar H. Saeed, Andrew W. Prentice, Gaia Neri, Chao Li, Adrian M. Gardner, Yang Bai, Reiner Sebastian Sprick, Igor V. Sazanovich, Andrew I. Cooper, Matthew J. Rosseinsky, Martijn A. Zwijnenburg, Alexander J. Cowan

**Affiliations:** † Stephenson Institute for Renewable Energy and Department of Chemistry, University of Liverpool, Liverpool L69 7ZF, U.K.; ‡ Department of Chemistry, University College London, 20 Gordon Street, London WC1H 0AJ, U.K.; § Department of Chemistry and Materials Innovation Factory, University of Liverpool, 51 Oxford Street, Liverpool L7 3NY, U.K.; ∥ Department of Pure and Applied Chemistry, University of Strathclyde, Thomas Graham Building, 295 Cathedral Street, Glasgow G1 1XL, U.K.; ⊥ Central Laser Facility, Research Complex at Harwell, STFC Rutherford Appleton Laboratory, Harwell Campus, Didcot, Oxfordshire OX11 0QX, U.K.

## Abstract

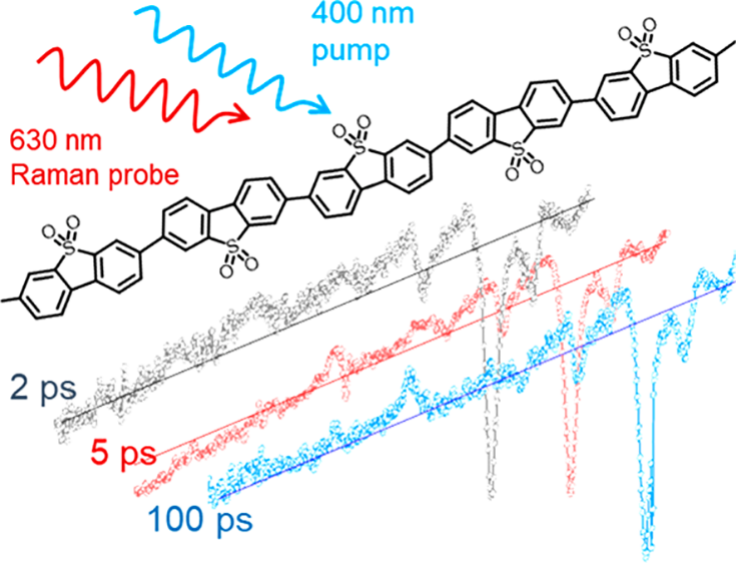

Polymer photocatalysts are a synthetically diverse class of materials that can be used for the production of solar fuels such as H_2_, but the underlying mechanisms by which they operate are poorly understood. Time-resolved vibrational spectroscopy provides a powerful structure-specific probe of photogenerated species. Here we report the use of time-resolved resonance Raman (TR^3^) spectroscopy to study the formation of polaron pairs and electron polarons in one of the most active linear polymer photocatalysts for H_2_ production, poly(dibenzo[*b*,*d*]thiophene sulfone), P10. We identify that polaron-pair formation prior to thermalization of the initially generated excited states is an important pathway for the generation of long-lived photoelectrons.

The development of scalable photocatalysts that can split water efficiently by using solar energy would transform the energy landscape, providing a way to generate hydrogen sustainably. Historically, research has focused on inorganic semiconductors, but in the past 12 years there has been a rapid increase in the study of organic photocatalysts for water splitting^1^ following studies in 2009 which showed that graphitic carbon nitride was an effective hydrogen evolution photocatalyst.^2^ More recently, a wider variety of classes of organic photocatalysts have been reported including polymeric networks such as conjugated microporous polymers (CMPs),^3^ covalent triazine-based frameworks (CTFs),^4−6^ covalent organic frameworks (COFs),^7−9^ and linear conjugated polymers.^10,11^ Among these, the linear homopolymer of dibenzo[*b*,*d*]thiophene sulfone (P10, Scheme 1) was shown to be one of the most active for hydrogen evolution, both when using a sacrificial electron donor^12^ and in a z-scheme water splitting system.^13^ P10 can also promote oxygen evolution^14^ and CO_2_ reduction,^15^ all under visible light irradiation.

**Scheme 1:**
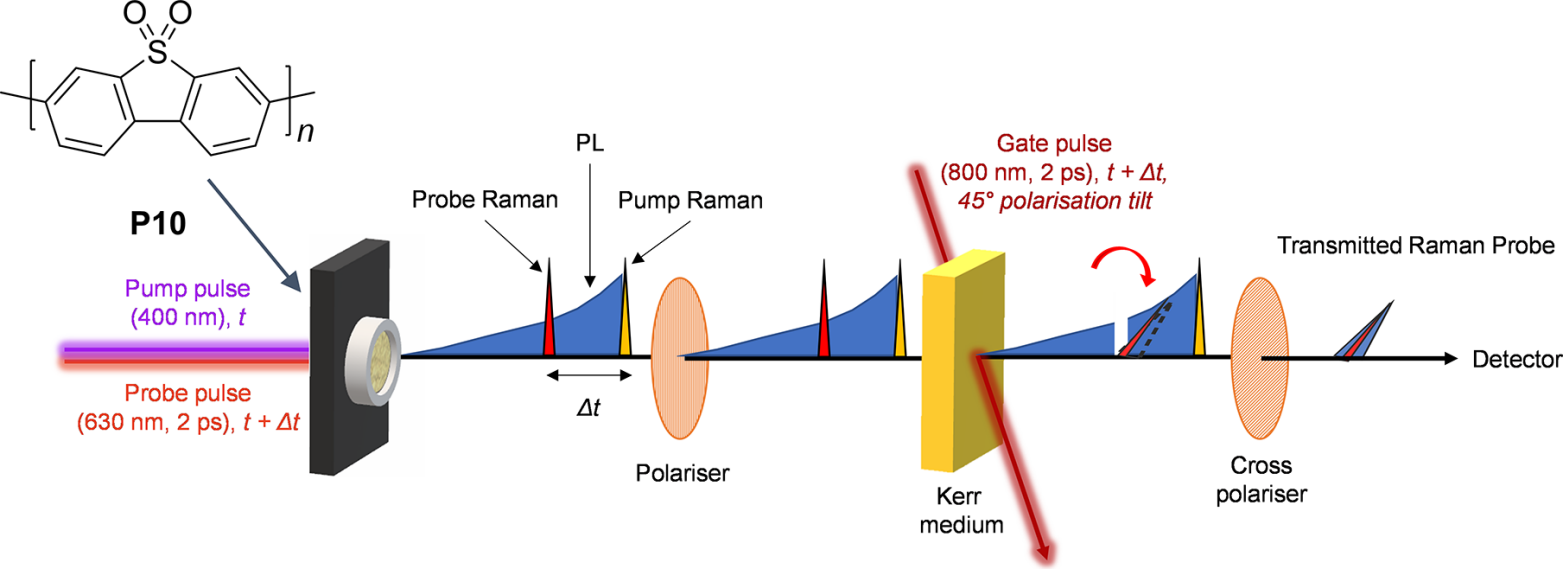
Kerr-Gated Time-Resolved Resonant Raman Experiment of P10 (Structure Upper Left) The laser pump pulse (400 nm) generates photoexcited states that can be subsequently interrogated by the probe pulse (630 nm) which is delayed with respect to the pump (Δ*t*). The wavelength of this probe pulse was selected to be resonant with an electronic transition associated with a transient species observed in the transient absorption spectra of P10. The probe pulse generates both Raman scatter and photoluminescence (PL). An ∼2 ps duration 800 nm high-energy laser pulse induced a transient optical anisotropy which lasts for approximately the duration of the gating pulse in the CS_2_ Kerr medium. During this period the gate is “opened”, and the polarization of the incident linearly polarized light is rotated by 90° with respect to its original orientation and passed through the crossed exit polarizer into the spectrometer while the unrotated light is rejected. Raman scattering is a fast process that occurs on a subpicosecond time scale. By synchronizing the timing of the Raman probe laser pulse with the gate pulse, it is possible to selectively transmit Raman scattered photons while rejecting the majority of the much longer lived (ns or greater) PL.

To facilitate the design of polymer photocatalysts and to truly exploit the synthetic control available, it is important to understand their underlying photophysics and mechanisms. In contrast to inorganic semiconductors, where the photogeneration of free charges occurs with a high efficiency, the poor dielectric screening of charges in organic absorbers means that polaron yields are often low; this is a central issue for this class of materials. Understanding why particular organic photocatalysts can efficiently generate separated charges following photon absorption is important. A body of literature exists on the underlying mechanisms of ultrafast polaron formation in organic photovoltaic (OPV) materials^16−19^ with proposed mechanisms including formation via hot and relaxed exciton dissociation and direct polaron-pair photogeneration. However, it is not clear if such models are also directly applicable to polymer photocatalysts where the additional presence of metal catalysts (e.g., for H_2_ and O_2_ evolution) and water may play an important role.

Transient absorption (TA) UV–vis spectroscopy is an established technique that has been widely applied to study electron–hole dynamics of inorganic and organic solar fuel materials.^20,21^ TA studies of P10^12−14,22,23^ report initial formation of a broad positive absorption at >700 nm assigned to a singlet excitonic state that decays on the picosecond time scale.^12^ In the presence of a sacrificial electron donor (commonly triethylamine (TEA) in a methanol/water solvent, 1:1:1 vol), a long-lived band at 630 nm has been assigned to an electron polaron (P10(e^–^)), proposed to form by quenching of the excitonic state by TEA on a time scale between 1 and 100 ps.^23,12^ In the absence of a sacrificial electron donor, a 630 nm TA band is still observed, and this has been proposed to be due to a polaron pair, a spatially separated weakly interacting electron and hole, with similar spectral characteristic to the P10(e^–^).^12,13^ The P10(e^–^) is very stable as it is retained on the polymer chain for ∼100 μs, despite the presence of residual Pd in the structure from the polymer synthesis which acts as a hydrogen evolution catalyst.^23^ For P10, fast polaron formation and trapping of the electron on the polymer leads to a high level of photocatalytic activity, but the chemical nature and mechanism of polaron formation are unclear.

Interpretation and assignment of TA features can be challenging due to the number of broad, often overlapped UV–vis bands. Time-resolved resonance Raman (TR^3^) spectroscopy directly probes the vibrational modes of short-lived intermediates, enabling assignment of nonequilibrium structures. Raman modes are sensitive to both the local structure and the intermolecular ordering of polymers, making TR^3^ a potentially useful way to study the mechanism and site of polaron formation.^24^ Time-resolved Raman spectroscopy has been used to study exciton conformational changes and polaron formation in OPV materials but has not been previously applied to study polymer photocatalysts.^24−27^ Here we apply TR^3^ to study the mechanisms of P10 polaron formation.

The ground state Raman spectrum (600–1400 cm^–1^) of P10 powder (633 nm Raman probe) shows peaks at 1411, 1340, 1301, 1269, and 1145 cm^–1^ (Figure 1a) and a strong band at 1596 cm^–1^ (Figure S1) that is outside the spectral window used for the TR^3^ experiment. These bands are assigned to ring/carbon backbone modes except for 1145 cm^–1^, which has contributions from the sulfone mode through comparison to Raman spectra predicted by density functional theory (DFT) calculations (Supporting Information, Figure S2 and Table S1); see section 1.4 of the Supporting Information for details about the computational method employed. TA experiments performed on P10 aggregates, formed from a toluene suspension, are shown in Figure S4. Toluene is used as an inert, nonpolar solvent to generate a thin layer of P10 for our TA experiment. In agreement with past reports of P10 in polar solvents,^12^ the TA spectra of the aggregates recorded following 400 nm excitation show a weak transient band between 620 and 660 nm, assignable to either a P10 polaron pair or the P10(e^–^) polaron. This assignment is also supported by the species associated spectra generated through target analysis fitting of the TA data, based on the kinetic model derived within this Letter as a result of the TR^3^ data, Figure S5, and the accompanying text. The TR^3^ spectra of P10 powder following 400 nm excitation recorded by using a 630 nm Raman probe that is resonant with the proposed P10 polaron are shown in Figure 1b–e. In common with many organic photocatalysts P10 is photoluminescent following excitation at energies greater than the optical gap (2.61 eV, λ < 475 nm; see Figure S3).^12^ Here we make use of an optically pumped Kerr gate to remove the majority of the photoluminescence (PL) background that otherwise masks the weak Raman scatter from the photogenerated transients (Scheme 1).^28−30^ 2 ps after excitation of P10 the TR^3^ spectrum shows bleaching (a decrease in scattering intensity) of the ground state Raman modes of P10, and only broad excited state Raman bands are present, which are assigned to vibrationally hot photogenerated state(s) (Figure 1b). Within 5 ps these begin to cool, and transient Raman bands are observed. These are centered at 713, 847, and 988 cm^–1^ (weak) and in the region of 1210 and 1110 cm^–1^ (partially overlapped with the ground state bleaches). The new transient features persist for longer than 1 ns (Figure 1e).

**Figure 1:**
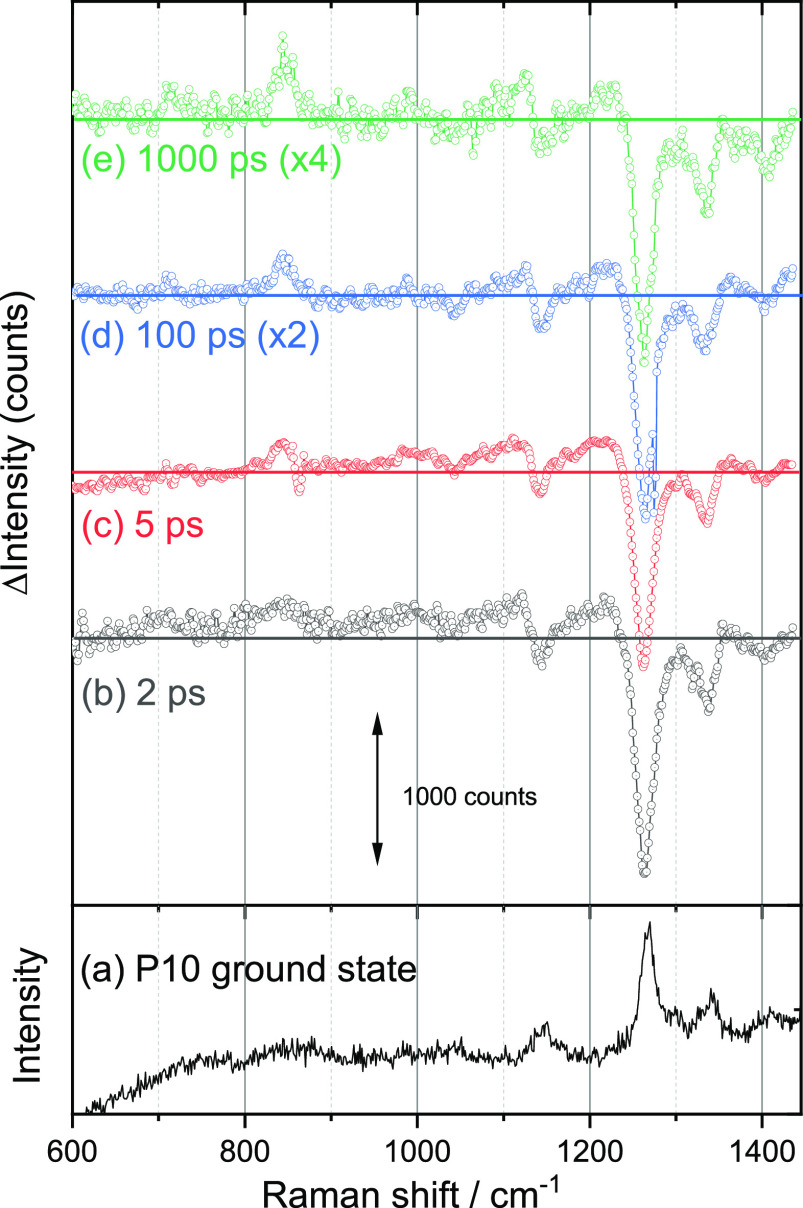
(a) Ground state Raman spectrum (633 nm probe) of P10 powder. (b–e) TR^3^ spectra of P10 recorded at the time indicated after 400 nm excitation of P10 powder by using a 630 nm Raman probe.

Two experiments were performed to test the assignment of the transient Raman bands to either P10(e^–^) or the polaron pair. First, we recorded TR^3^ spectra in the presence of a sacrificial electron donor (TEA/methanol/water, 1:1:1) that will increase the yield and lifetime of the P10(e^–^) polaron. For a discussion of the implications of P10(e^–^) accumulation on the TR^3^ experiment, please refer to the text accompanying Figure S6. The TR^3^ spectra show transient bands at 719, 849, 1138, 1262, and 1331 cm^–1^, in good agreement with the TR^3^ data recorded in the absence of the sacrificial electron donor (Figure 1 and Figure S6). The TR^3^ bands at 1262 and 1331 cm^–1^ were not visible in the absence of the electron donor (Figure 1) due to the overlap with the P10 ground state bleach. Second, as P10(e^–^) accumulates under steady state illumination,^23^ we have also recorded the Raman spectra under 365 nm LED illumination using a conventional (steady state) microscope both on resonance (633 nm) and off resonance (532 nm) with the known UV–vis absorption maximum of P10(e^–^) (Figure 2a, b). The Raman spectrum of the photogenerated P10(e^–^) by using a 633 nm probe wavelength shows excellent agreement with the proposed P10(e^–^) TR^3^ spectrum (Figure 2c), while the spectrum recorded with a 532 nm probe under identical conditions shows no bands that can be assigned to a photogenerated species.

**Figure 2:**
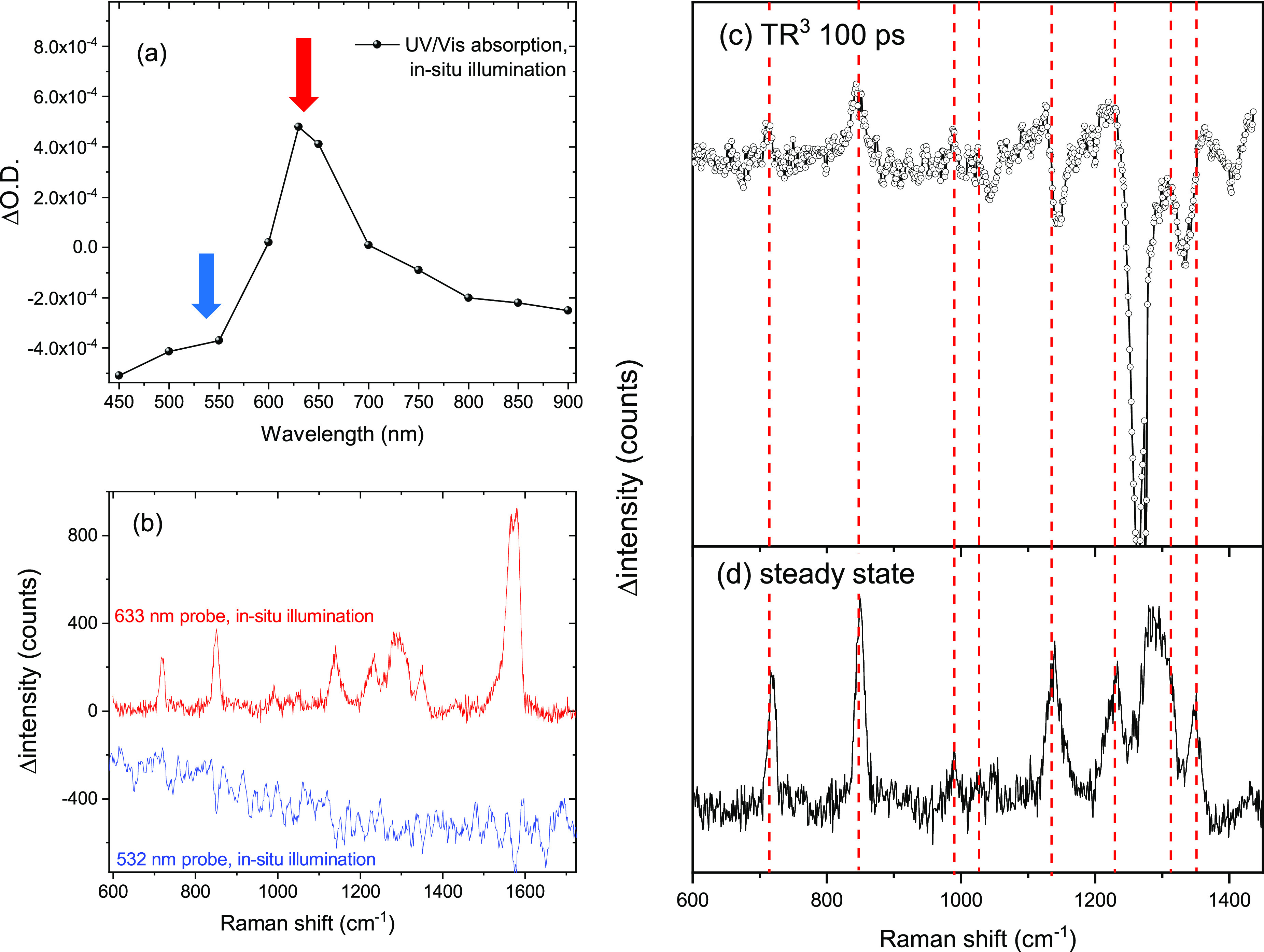
(a) UV–vis spectrum of P10(e^–^) generated by CW 365 nm illumination of P10 in the presence of TEA/methanol/water (1:1:1). The red and blue arrows indicate the Raman probe wavelengths used in (b). (b) Raman difference spectrum (using the sample in the dark as a background) of P10 in the presence of TEA/methanol/water (1:1:1) on and off resonance with the observed feature in the UV–vis spectrum. (c, d) Comparison between the steady state (d) and TR^3^ Raman data (c, 100 ps) which show the presence of the same species.

Preresonance^31^ and resonance^32^ Raman spectra for P10, P10(e^–^), and one-electron-oxidized P10 (P10(h^+^)) of the monomer and oligomers of different length have been predicted by DFT using the ωB97XD exchange-correlation functional^33^ and the cc-pVDZ basis set.^34,35^ See Figure 3 for a comparison between the experimental spectra and predicted (pre)resonance spectra for a P10 hexamer and Figures S7–S10 for all predicted (pre)resonance spectra for the different oligomer lengths and those predicted using another functional. All DFT predicted spectra discussed herein and within the Supporting Information have been scaled by the same factor, obtained by aligning the intense predicted P10 peak to that of experiment. For all oligomer lengths the predicted P10 preresonance Raman spectrum is dominated by a single intense transition with good agreement to its experimental counterpart (see Figure 3). The predicted resonance Raman spectra for P10(e^–^) and P10(h^+^) show an increased number of intense peaks below 1600 cm^–1^ which differ depending on the excited state the probe wavelength is on resonance with, complicating our ability to distinguish between the two species. However, for oligomers it is apparent that the calculations in the case of P10(e^–^) consistently reproduce the experimentally observed red-shift of the strong 1596 cm^–1^ peak of the P10 polaron species. This is not the case for its P10(h^+^) counterparts for which the intense peak is predicted to be unshifted relative to that of P10. Resonance Raman spectra predicted with a different range-separated exchange-correlation functional, CAM-B3LYP (see Figure S9) suggest that this red-shift of the most intense peak in the Raman spectra is representative of P10(e^–^) oligomers and the lack of such a shift typical of their P10(h^+^) counterparts. In Figure 3, the unpaired electron density is shown for the P10(e^–^) and P10(h^+^) hexamer species. For both species, the polaron is localized on the central P10 moieties, with the calculations of P10(e^–^) showing increased electron density on the thiophene ring.

**Figure 3:**
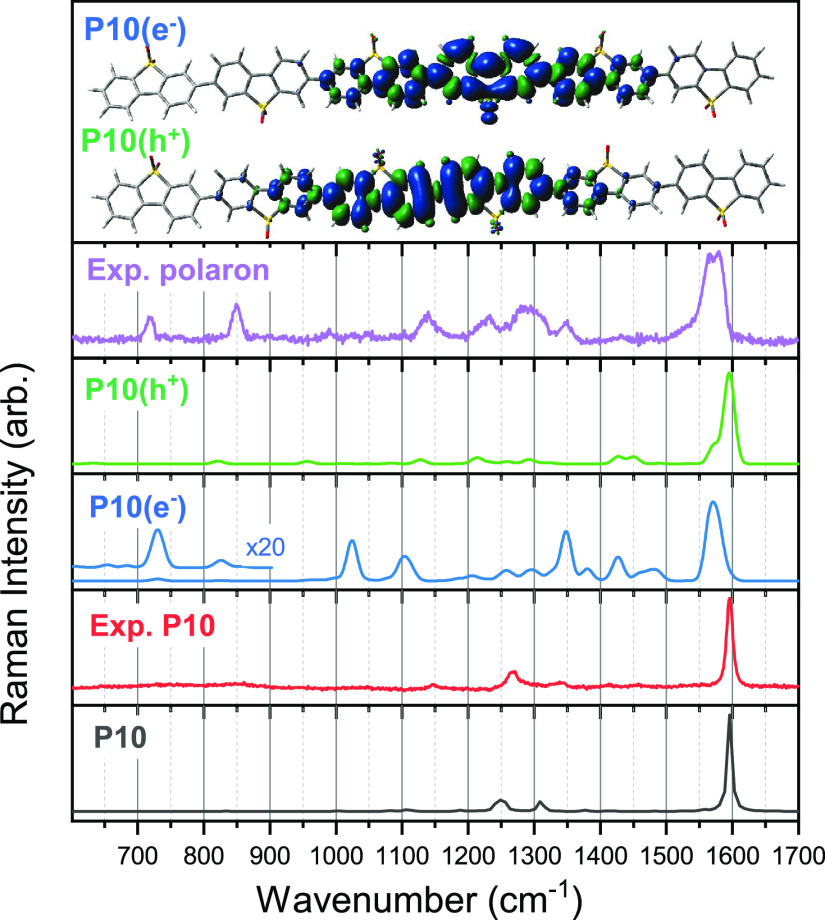
Comparison of the experimental spectra of P10 and the P10 polaron to the predicted (pre)resonance (ωB97XD/cc-pVDZ) spectra of a P10 hexamer in different charge states. All predicted spectra have been scaled by a factor of 0.943, obtained by aligning the intense peak in the predicted neutral spectrum to that of the P10 experimental spectrum. The predicted resonance Raman spectra shown for P10(e^–^) and P10(h^+^) are calculated for the intermediate excited state with a predicted vertical absorption closest to 630 nm. Similar spectra for other intermediate excited states can be found in Figure S10. The inset is a schematic of the polaron localization for the two charged species.

These results indicate that by using the TR^3^ experiment we are measuring the spectrum of either the P10(e^–^) polaron or the vibrational modes associated with electron localization within a polaron pair. We now turn to the rate and mechanism of polaron formation in the absence and presence of a sacrificial electron donor (Figure 4). An advantage of the TR^3^ experiment is that contributions from other off-resonance intermediates are minimal, simplifying the analysis of the transient data. The kinetics of the 847 cm^–1^ Raman band are studied, but all TR^3^ bands in this spectral region show similar kinetics. Following excitation of P10 in the absence of a sacrificial electron donor the decay can be well fitted to a biexponential function with an initial rise in intensity, which is close to the instrument response function (τ ∼ 3 ps), and a subsequent slower decay (101 ps) to form a population that persists until the longest time scales studied (3.4 ns); full fitting parameters are in Table S5. The slow decay is not due to electron transfer from the P10 to residual Pd left during polymer synthesis, as this is known to occur on the microsecond and slower time scale;^23^ instead, it is assigned to recombination of the polaron pair. The fast rise in intensity of the 847 cm^–1^ band is in line with the observed rate of vibrational cooling (Figure S11), from which we estimate that the transient species reach thermal equilibrium by ∼10 ps. After 10 ps, we see no further increase in intensity of the 847 cm^–1^ Raman mode (Figure 4). To probe the lifetime of the excitonic state, PL between 634 and 697 nm is measured by blocking the Raman probe beam during the Kerr gated experiment (Figure S12). The PL at 657 nm has an amplitude-weighted average lifetime of 314 ps (Table S6), demonstrating that the excitonic state of P10 persists at time scales beyond those where we observe polaron formation. This leads to the conclusion that polaron formation does not occur at significant levels from the thermalized exciton state, despite this species persisting for several hundred picoseconds. Instead, the thermalized exciton decays to the ground state, resulting in the slow recovery of the negative P10 band in the TR^3^ spectrum at 1265 cm^–1^ (Figure S13). In the time-resolved experiment P10 is excited with photon energies ca. 0.5 eV greater than the P10 optical gap;^12^ this excess energy is important in enabling P10(e^–^)/polaron pair formation, with hot exciton dissociation being the dominant pathway for P10 in the absence of a sacrificial electron donor. Target analysis of the TA data (Figure S5 and accompanying text) shows that the TA data can be well fitted to the TR^3^-derived model with ∼95% of the polaron-pair population being generated with a lifetime of ∼4.5 ps directly from the hot excitonic state.

**Figure 4:**
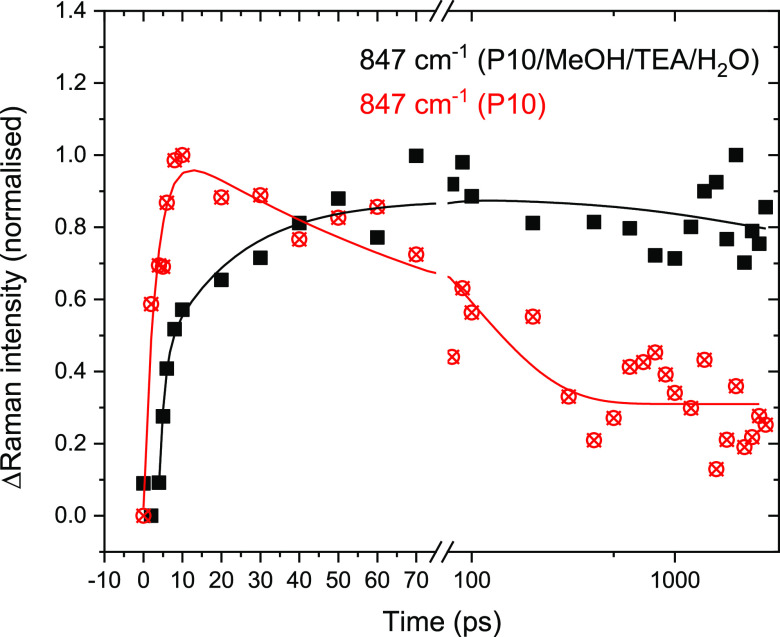
(a) Kinetics of P10 polaron Raman band at 847 cm^–1^ following 400 nm excitation. P10 powder (no electron donor) data are shown with red circles, and data with a MeOH/TEA/H_2_O sacrificial electron donor are shown with black squares. The parameters of the fit lines are described in the text and Table S5. The data are normalized (to 0, 1) to allow easy comparison to the Raman kinetics of P10 in the absence of the sacrificial electron donor. The kinetics of the 847 cm^–1^ bands are baseline corrected by taking the difference in Raman scatter at 847 and 928 cm^–1^, where no transient bands are present.

In the presence of the sacrificial electron donor the P10(e^–^) Raman band (847 cm^–1^, Figure 4) shows both a fast initial rise (τ < 2 ps) and a second slower growth (τ ∼ 20 ps). The similar fast lifetime component in the presence and absence of an electron donor shows that polaron formation occurs both directly from the hot excitonic state (<2 ps) that is present for up to 10 ps (Figure S11) and via reductive quenching of the thermalized exciton by the amine electron donor (∼20 ps), with both processes contributing similar amounts to the overall amplitude (Table S5). We see only minimal decay of the 847 cm^–1^ band in the presence of the sacrificial electron donor, suggesting that hole transfer is occurring following the fast polaron-pair formation. The conclusion that reductive quenching of the relaxed excitonic state can occur is supported by PL measurements at 657 nm which show a decreased amplitude weighted lifetime (67 ps) with a fast decay component (τ ∼ 13 ps) in the presence of the electron donor mix (Table S6 and Figure S13). A significant PL population persists to >100 ps, showing that a proportion of the exciton population is inaccessible to the scavenger. Past studies have correlated the yield of P10(e^–^) to the driving force for hole transfer from the polymer exciton to a sacrificial electron donor,^12^ and we confirm the presence of this pathway. However, the observation that a similar fast rise in the 847 cm^–1^ band is existent in both the presence and absence of the electron donor suggests that fast exciton dissociation to form polaron pairs with subsequent electron transfer from the sacrificial electron donor is also occurring, and this is a major contributing factor to the high level of measured photocatalytic activity of P10 for hydrogen evolution.

More widely, hot exciton dissociation is expected to be of particular importance for other particulate polymer photocatalysts which exist as aggregates ranging from several hundred nanometers to micrometers.^12^ Most photons will be absorbed away from the polymer/solvent (water) interface. In addition to preventing access to the sacrificial electron donor, the absence of the high-dielectric environment presents a large barrier to dissociation, with past calculations^36^ of binding energies of ∼1.2 eV for excitons within the polymer matrix of a similar linear polymer, as compared to only ∼0.17 eV near the polymer/water interface. In the absence of charge separation occurring prior to thermalization driven by the excess energy of the hot exciton, most excitons formed away from the polymer/interface would be lost via parasitical de-excitation.

In conclusion, we have shown that Kerr gated TR^3^ spectroscopy enables the study of ultrafast polaron electron formation in P10, a highly active hydrogen evolution photocatalyst under sacrificial conditions. More widely, we propose that it is a valuable technique for the study of photogenerated transients of polymer photocatalysts and photoelectrodes and could contribute to the effective design, for example, of Z-scheme composite materials for overall water splitting. A combination of experimental Raman spectroscopy and DFT calculations supports the assignment of the TR^3^ spectra, and the calculations indicate that the electron is predominantly localized on a single P10 moiety, in particular around the thiophene ring, which is beneficial given the proposed role of the sulfone groups in enabling water molecules to localize providing a more polar environment.^12^ Past models have focused on P10(e^–^) formation through hole transfer from the excitonic state to the sacrificial amine.^12^ Here we also show that hole transfer following polaron-pair formation from hot states is also an important pathway for forming long-lived P10(e^–^). It is known from OPV research that the distribution of excess energy following singlet exciton formation can have a critical role in facilitating charge separation,^18^ and our work also demonstrates the importance of polaron pair formation prior to thermalization for this polymer photocatalyst, P10.

## Supporting Information Available

Experimental procedures, details of the DFT calculations of P10 with different chain lengths, transient absorption data and additional TR^3^ data (PDF)
DFT optimized geometries of all relevant structures (ZIP)
