# Supplementary material for: Time-Resolved Raman Spectroscopy of Polaron Formation in a Polymer Photocatalyst
Source: J Phys Chem Lett. 2021 Nov 3;12(44):10899–905. doi: 10.1021/acs.jpclett.1c03073 (PMC8591663; doi:10.1021/acs.jpclett.1c03073)
Supplement: Supplementary file 1 — jz1c03073_si_001.pdf [file jz1c03073_si_001.pdf]

Supporting information

Time-Resolved Raman Spectroscopy of Polaron Formation in a Polymer Photocatalyst

Verity L. Piercy,[‡,a] Khezar H. Saeed,[‡a] Andrew W. Prentice,[‡b] Gaia Neri,[a] Chao Li[a,c], Adrian M. Gardner,[a] Yang Bai,[c] Reiner Sebastian Sprick,[c,d] Igor V. Sazanovich,[e] Andrew I. Cooper,[c] Matthew J. Rosseinsky,[c] Martijn A. Zwijnenburg,[b] and Alexander J. Cowan[a]

1. Experimental details:

Polymer P10 was prepared uisng Suzuki-Miyaura-type polycondensation:[1] A flask was charged with the 3,7-dibromodibenzo[*b*,*d*]thiophene 5,5-dioxide (281 mg, 0.75 mmol), 3,7-bis(4,4,5,5-tetramethyl-1,3,2-dioxaborolan-2-yl)dibenzo[*b*,*d*]thiophene 5,5-dioxide (351 mg, 0.75 mmol), *N*,*N*-dimethylformamide (20 mL), an aqueous solution of K_2CO_3 (4 mL, 2.0 M), and $[Pd(PPh_3)_4]$ (15 mg). The mixture was degassed by bubbling with N_2 for 30 minutes and heated to 150 °C for 48 hours. The mixture was cooled to room temperature and poured into water. The precipitate was collected by filtration and washed with water and methanol. Further purification of the polymers was carried out by Soxhlet extraction for 2 days with chloroform to remove any low-molecular weight by-products. The product was dried under reduced pressure at 80 ^{o}C and obtained as a yellow powder (290 mg, 90%). Residual palladium content was determined to be 0.4% via ICP-OES. Methanol (≥99.8%, Sigma Aldrich), TEA (BioUltra, ≥99.5%), were used as recieved.

1.1 Kerr-gated Time-Resolved Resonance Raman (TR^3) specterocopy

TR^3 experiments were carried out at the UK Central Laser Faccility using the ULTRA apparatus.[2] A schematic of the TR^3 experiment can be found in the main paper (scheme 1) [3–5] Raman scattering of P10 was induced by a 630 nm laser pulse (4 mW, 10 kHz, 2 ps) with a spot size of 75 x 75 μm and excitation of the sample was achieved using a 400 nm pump pulse (5 mW, 10 kHz, 2 ps) with a spot size of 100 x 100 μm, which were overlapped on the

front face of the sample. The Raman and PL signals were collimated and passed through a long pass filter (633 nm, Semrock RazorEdge LP02-633RE), which acts to block Rayleigh scattering, before reaching the Kerr-gate. The Kerr-gate consists of a Kerr-medium (2 mm path length quartz cell filled with CS_2) and 2 crossed polarisers (25 mm aperture, Halbo Optics PS25). Activation of the Kerr-gate was achieved using a gating pulse (800 nm, 240 μJ, 2 ps, 2.4 W, 10 kHz), which was produced by a ps arm of the ULTRA Laser System and was polarised at 45 ° with respect to the polarisers and was focused before the Kerr-medium. When the Kerr gate is active, the rotated polarization of the Raman signal enables it to pass through the output polariser, whislt the majority of the PL (the polarization of which has not been changed) is rejected. The 770 nm short-pass filter (Semrock FF01-770/SP) was placed after Kerr gate to block the scatter of 800 nm gating beam. The Raman scatter was disperesed via a spectrograph (Andor Shamrock 303i) and detected using a CCD camera (iDus DU-420A-BU2, Andor). Timing of the gating, probe and pump pulses was changed using optical delay lines (IMS-600LM, 600 mm travel range and UTS100CC, 100 mm travel range, both from Newport). The Raman shift was calibrated against the Raman spectra obtained for toluene and acetonitrile

Spectra were collected and averaged over 3 repeats, each with an acquisition time of between 20 s (for short runs to generate kinetic traces) and 60 s (for generation of higher signal:noise spectra). Delay times were randomised during each scan to minimise systematic effects. For experiments using a water:methanol:triethylamine solvent the sample was replaced periodically due to the build up of gas bubbles and a small number of delay times (≤ 16) was studied with each sample. Samples were prepared by sandwiching dry powders or powders with a drop of the water:methanol:triethylamine mixture between two CaF_2 windows with a 300 μm spacer and loaded into a Harrick cell which was purged with N_2 for about an hour before measurements were taken. Samples were measured in a near back-scattering geometry

and the sample was continually rastered to minimise the effect of photoproduct build-up during the experiment.

The Kerr gate was successful in removing the majority of the pump (400 nm) induced PL, but there was still some present in the recorded spectra, see scheme 1 main text. To correct spectra (and to provide time-resolved PL data) a series of experiments was also recorded where the Raman probe laser pulse arrived 100 ps prior to the 400 nm pump and 800 nm Kerr gate pulses. These spectra were then subtracted from the TR^3 data at each gating pulse time delay using a home-made routine in Labview (National Instruments) that also generated the Raman difference spectra.

1.2 Transient absorption (TA) spectroscopy

TA spectra were recorded using a Harpia-TA spectrometer (Light Conversion) in an experimental configuration reported previously.[6] Briefly the system consists of a femtosecond laser syetsm (Pharos-SP-10W, Light Conversion) that has an output wavelength of 1028 nm and a pulse duration of ~180 fs at 10 kHz. 1 W of the 1028 nm output is used to generate 400 nm excitation pulses using a commercial OPA with second harmonic generation module (Orpheus and Lyra, Light Conversion). Experiments were carried out with a 400 nm excitation (beam diameter *ca.* 600 μm at sample, power at sample ~ 500 μW, effective pumping repetition rate of 5 kHz). The white light TA probe beam is generated by focusing 1028 nm light onto a CaF_2 crystal within the Harpia spectrometer and focussed to *ca.* 400 μm at the sample. The samples of P10 (2.4 mg) were suspended in toluene (10 ml). The solution was purged with Ar for 10 minutes, and then placed into a quartz cuvette (pathlength of 2 mm) which was sealed with a septa and flushed with Ar prior to filling. A thin film of aggregates formed on the quartz cuvette walls. The difference in probe beam transmission in the pumped/un-pumped state was measuredusing a NMOS detector (S3901, Hamamatsu),

following dispersion by a spectrograpgh (Kymera 193i, Andor). Data was analysed using Carpetview software (Light Conversion).

1.3 Raman Microscopy/steady state details

Steady state Raman spectra were recorded on a Renishaw InVia Confocal Raman Microscope, using either 633 nm (~1.5 μm spot size at 0.875 mW) or 532 nm (~1.3 μm spot size at 2.5 mW) Raman laser probes. P10 powder was sandwiched between two CaF2 windows and wetted with a few drops of a 1:1:1 mixure of water:methanol:triethylamine. Spectral acquisition involved 2 accumulations at 10 s exposure time. A 365 nm LED was mounted to the stage of the Raman microscope to illuminate the sample with a power of ~15 mW cm^{-2} during Raman acquisition. No significant background from scattered 365nm LED light was detected in the Raman spectra, allowing direct subtraction of spectra with and without illumination at the two laser wavelengths to plot the difference spectra shown in Figure 2b.

1.4 Computational details

All density functional theory (DFT)[7,8] calculations were performed with Gaussian 16 (Revision A.03).[9] All structures correspond to minimum energy arrangements i.e., stationary points on the multi-dimensional potential energy surface, and subsequently validated through analytic computation of the hessian matrix, showing positive curvature along all $3n$-6 vibrational modes, where n represents the number of atoms within the system. Unless stated otherwise an implicit solvent model was employed, specifically the integral equation formalism polarizable continuum model (IEF-PCM),[10–13] with the dielectric constant set to 78.36, representing bulk water.

Preresonance Raman spectra for the neutral oligomers where the Raman excitation wavelength is much longer than the wavelength corresponding to the absorption onset were obtained by specifying the “Raman” option for the frequency keyword in Gaussian 16 in

combination with adding the "ROA" option for the polar keyword and the wavelength of the probe (630 nm).[14] Resonance Raman spectra were calculated using the FC option in Gaussian 16 and involved the Frank-Condon approximation for the transition dipole, as well as the vertical gradient approximation.[15] Resonance Raman spectra were calculated for excitation wavelengths corresponding to the lowest 4 or 5 vertical excitations of one-electron oxidized and reduced oligomers, as well as for the experimental wavelength of the probe. Finally, unless stated otherwise all DFT predicted (pre)resonance Raman spectra have been scaled by the same factor, obtained by aligning the most intense peak at ~1600 cm^{-1} in the experimental and predicted preresonance Raman neutral P10 spectra.

The following exchange-correlation functionals were used in the DFT calculations B3LYP,[16–18] CAM-B3LYP[19] and ωB97XD,[20] in-conjunction with a cc-pVDZ basis set. For B3LYP and CAM-B3LYP dispersion interactions were accounted for by inclusion of Grimme's D3 dispersion model,[21] while with ωB97XD by default accounting for these interactions through a version of Grimme's D2 dispersion model.[22] The time-dependent extension to DFT (TD-DFT)[23] was used for the prediction of vertical excited states and compared to that predicted by equation-of-motion coupled cluster singles and doubles (EOM-CCSD).[24–27] The chain length of P10 modeled is denoted by $P10_x$, where x is either 1, 3 or 6 representing the monomer, trimer and hexamer, respectively, while the one electron-reduced and one-electron oxidized species are denoted by (e^-) and (h^+), respectively.

Tables S2-S4 present the lowest vertical excited state energies (VEE) of the different charge-states of $P10_1$, calculated with EOM-CCSD and TD-DFT using different exchange-correlation functionals while figures S7-S10 show the Raman spectra of the different charge-states of $P10_1$, $P10_3$ and $P10_6$. A comparison of the EOM-CCSD and TD-DFT results suggests that the range-separated functionals, as expected, yield more similar vertical excitation energies to EOM-CCSD than those obtained by B3LYP. As the calculated vertical

excitation energies correspond to poles of the dynamic molecular polarizability and because the intensity of Raman peaks finds it origin in the change of this polarizability for a particular vibrational mode it stands to reason that the range-separated functionals should be preferred over B3LYP for prediction of the (pre)resonance Raman spectra, especially since we want to compare our prediction directly to experiment.

We observe an excellent agreement between the experimental neutral P10 Raman spectrum and the predicted preresonance Raman spectra for all oligomers and functionals, especially after scaling. For $P10_3(e^-)$ and $P10_6(e^-)$ we observe a shift of the strong peak at around 1600 cm^{-1} to lower wavenumbers, in line with the shift observed experimentally when comparing the neutral and polaron P10 spectra. This shift is not observed for $P10_3(h^+)$ and $P10_6(h^+)$ as there the strong peak remains in approximately the same place as for the neutral species. This shift in the case of $P10_3(e^-)$ and $P10_6(e^-)$ and the absence of such a shift for $P10_3(h^+)$ and $P10_6(h^+)$ is, in combination with the fact that such a shift is observed experimentally, probably the most compelling theoretical evidence that the experimental polaron signal is due to an electron rather than a hole polaron.

2. Supplementary data:

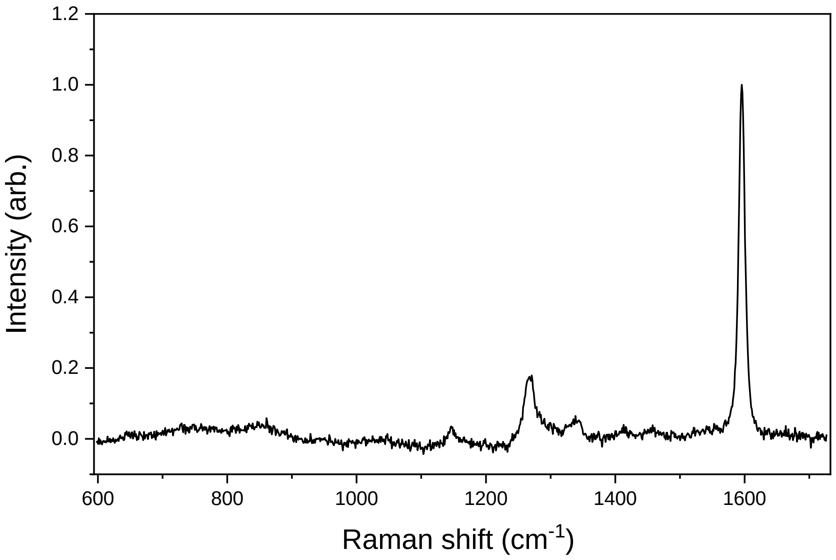

Figure S1. *Ground state Raman spectrum (633 nm) of P10 powder (full spectral window).*

Table S1 *The five most intense preresonance Raman peaks, predicted in the gas phase and up to 2000* cm^{-1}*. In addition to these peaks we provide information regarding to the first mode involving movement of the sulfone. The scaled values correspond to a scaling of 0.943.*

***P10**$_1$*		
Frequency (scaled)	*Raman Activity [arb.]*	*Characterization*
1688.01 (1591.79)	*650.99*	*carbon backbone*
1325.46 (1249.91)	*280.35*	*carbon backbone*
1535.14 (1447.64)	*131.88*	*carbon backbone*
1063.02 (1002.43)	*75.50*	*carbon backbone*
1255.46 (1183.9)	*68.23*	*carbon backbone*
1177.21 (1110.11)	*68.13*	*sulfone*
***P10**$_3$*		
1695.05 (1598.43)	*51513.85*	*carbon backbone*
1333.18 (1257.19)	*6694.38*	*carbon backbone*
1388.47 (1309.33)	*5083.56*	*carbon backbone*
1324.92 (1249.4)	*3587.29*	*carbon backbone*
1307.66 (1233.12)	*2259.70*	*carbon backbone*
1175.03 (1108.05)	*851.13*	*sulfone*
***P10**$_6$*		
1694.54 (1597.95)	*194559.50*	*carbon backbone*
1695.49 (1598.85)	*46796.55*	*carbon backbone*
1389.23 (1310.04)	*28871.66*	*carbon backbone*
1317.71 (1242.6)	*14028.58*	*carbon backbone*
1332.17 (1256.24)	*11805.55*	*carbon backbone*
1164.47 (1098.1)	*2075.2765*	*sulfone*

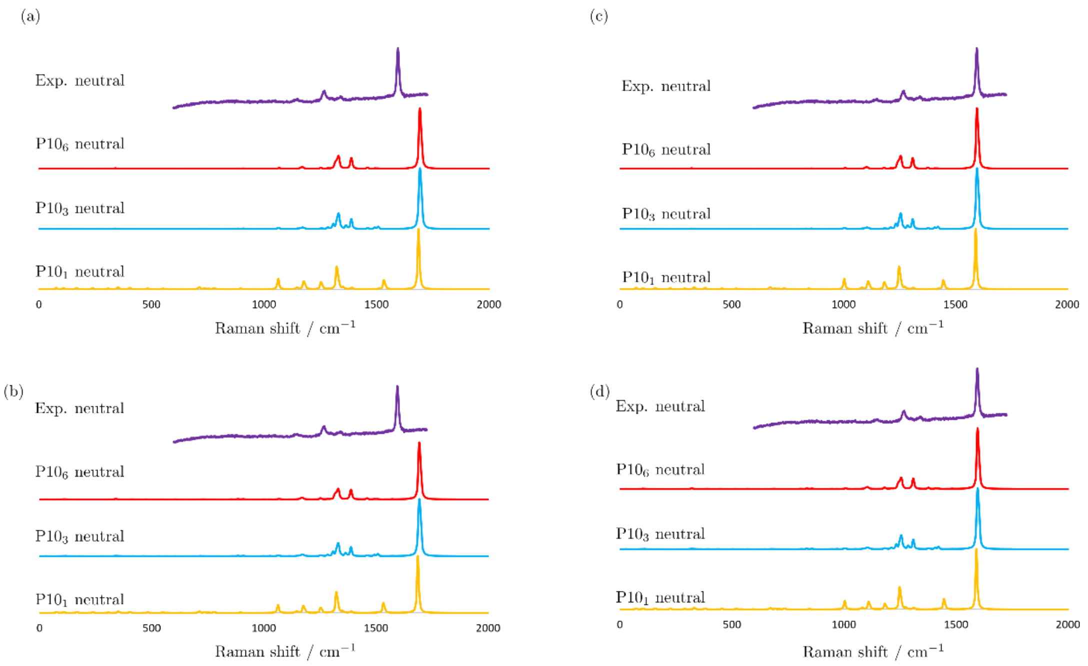

Figure S2. *The unscaled predicted gas phase Raman (a) and preresonance Raman (b) of neutral P10 oligomers. The scaled predicted gas phase Raman (c) and preresonance Raman (630 nm) (d) of neutral P10 oligomers, a scaling factor of 0.943 was used. All spectra were predicted using the ωB97XD exchange-correlation functional and the cc-pVDZ basis set, with a peak half-width at half height of 10* cm^{-1}*. The predicted intensities of each oligomer are normalized to the most intense signal in each spectrum.*

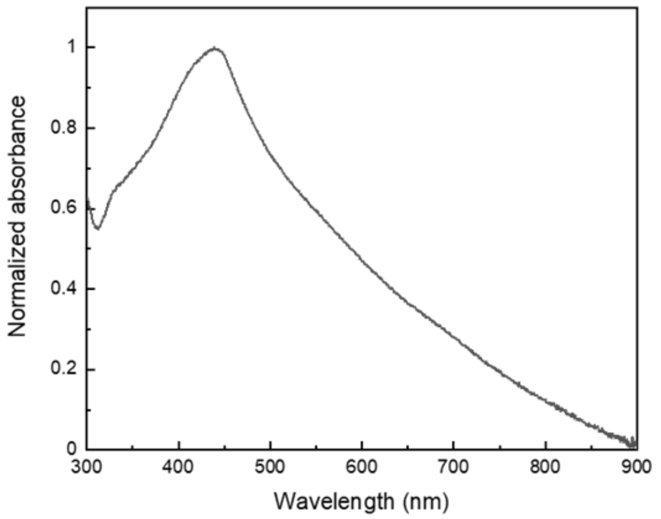

Figure S3*. P10 ground state UV-Vis spectrum*

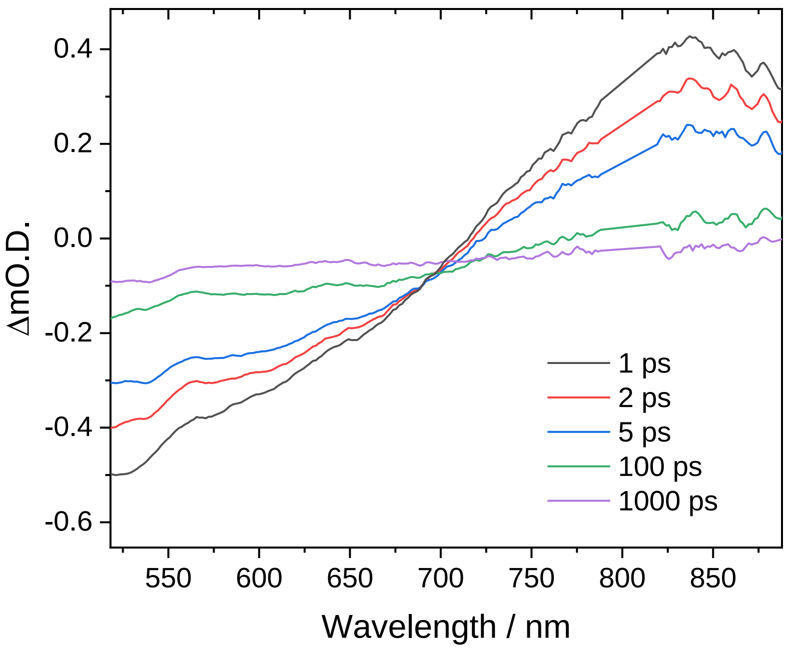

Figure S4. *Transient absorption (TA) spectra of P10 aggregates recorded following 400 nm excitation. The TA spectra of P10 aggregates suspended in toluene show a broad photoinduced absorption at wavelengths > 700 nm due to the formation of a singlet exciton. The TA spectra show a broad negative signal from < 500 – 700 nm due to stimulated emission from the excitonic state. A transient band at 620-660 nm is overlapped with this broad bleach and appears as a small absorption on a larger negative feature, hence the overall signal is negative and this is assigned to the polaron state in-line with past reports of P10 TA spectra (Nat. Commun. 2018, 9 (1), 4968). The feature at ~525 to 575 nm is not assigned but it may arise from structure within the stimulated emission bands, future experiments will explore this result.*

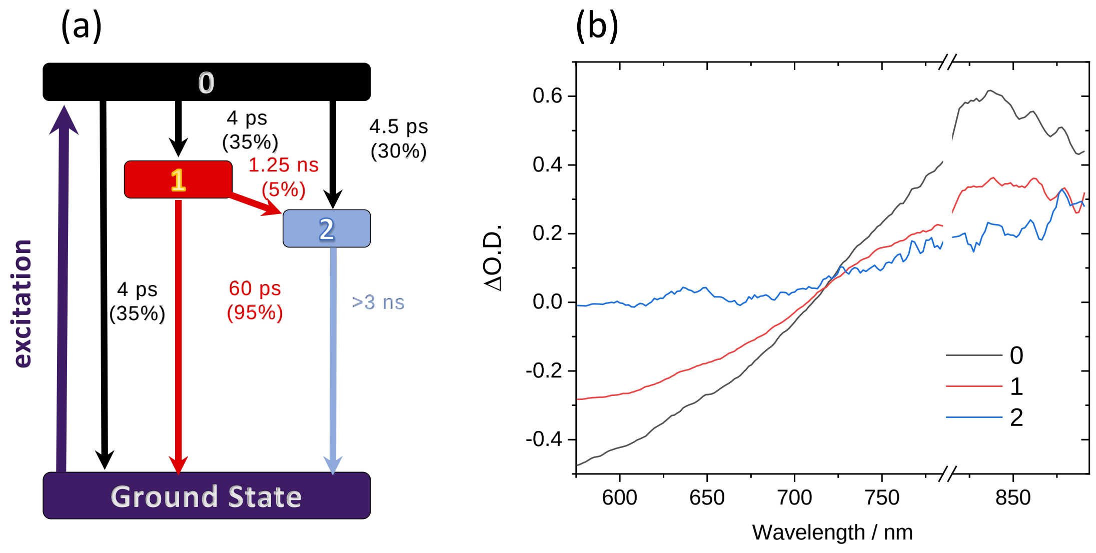

Figure S5. *Target analysis of TA data of P10 aggregates in an inert environment (a) shows the kinetic models (described below) used with fitting lifetimes. (b) is the resultant species associated spectra (SAS)*

Monitoring the kinetics of the polaron-pair (620-660 nm) from the unprocessed TA data (figure S4) is complicated by the presence of larger broad stimulated emission peaks (centered *ca.* 523 and 603 nm) which have decay at a different rate. However from the information obtained using TR^3 we construct a kinetic model for polaron formation, as described in the main text that can be used to fit the TA data (IRF ~170 fs). In the model the initially generated hot exciton state (compartment 0) that can either relax to the ground state, a thermalized excitonic state (compartment 1) or form a P10(e^-)/polaron pair. We also allow for polaron generation from the lower energy excitonic state (1), figure S5. We fit using a target analysis module within Carpetview software package (Light Conversion), where the transitions between compartments are modelled as single exponential functions that are convoluted by the instrument response function. A full description of the principles of target fitting of TA decay data can be found elsewhere.[28]

The species associated spectra (SAS) generated for P10 aggregates in toluene are shown in figure S5b and they align with the proposed kinetic models. The SAS of compartments 1 and 2 both show stimulated emission peaks (< 600 nm) and a broad absorption assignable to a singlet exciton, but with differing peak widths and relative intensities, in-line with species 0 being an initially generated hot excitonic state. The SAS of compartment 2 shows a peak at *ca.* 642 nm is seen in the toluene/P10 data which is assigned to the P10 polaron pair, in excellent agreement with a past TA study on this polymer photocatalyst.[1] The approximate lifetimes for each pathway are shown in figure 5a and the fitting supports the conclusion that the P10 polaron pair is primarily formed an initially generated hot state (compartment 0 to 1), with minimal contribution from the relaxed exciton (compartment 1 to 2). There is very good agreement between the lifetimes identified through fitting of the TR^3 data (*ca.* 3 ps) and the TA data (~ 4.5 ps) for the generation of the polaron pair. Once the system relaxes to the lower energy excitonic state (τ ~ 4 ps) relaxation to the ground state is the dominant pathway (95%).

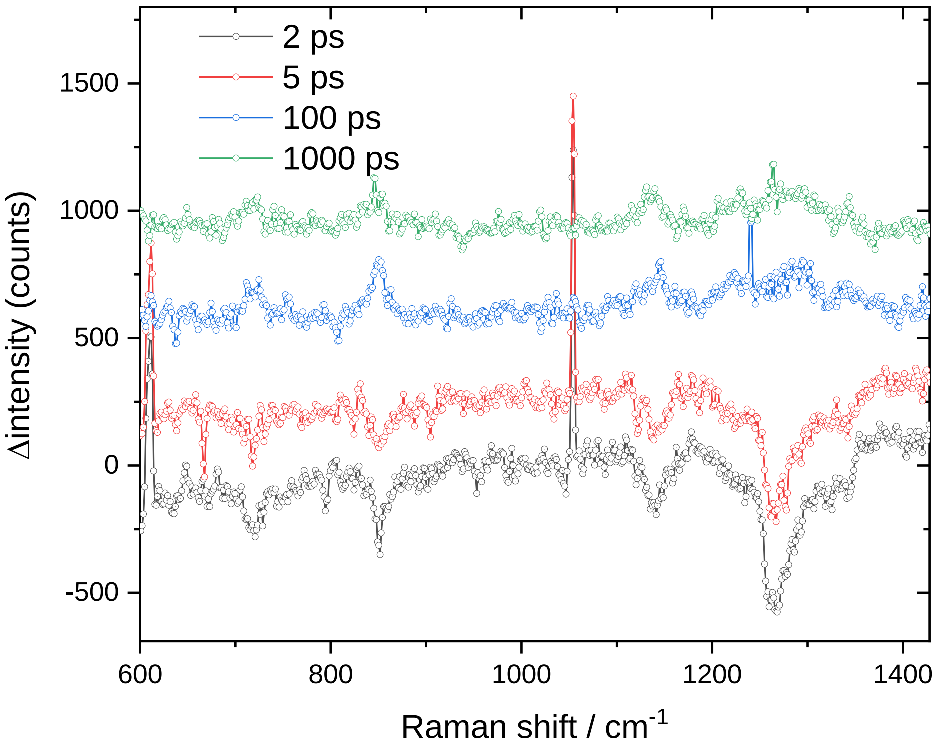

Figure S6. *TR*^{3} *spectra of P10 in the presence of a sacrificial electron donor (*$CH_3OH/TEA/H_2O$*, 1:1:1) recorded at the time indicated after 400 nm excitation of P10 powder using a 630 nm Raman probe.*

TR^3 difference spectra of P10 in the presence of a sacrificial electron donor ($CH_3OH/TEA/H_2O$, 1:1:1) show negative bands (bleaches) at early time delays as during the experiment the population of long-lived P10(e$^-$) accumulates and can itself undergo subsequent excitation by the 400 nm pump pulse. The negative bands at 719, 847, 1138, 1264 and 1331 cm^{-1} are in good agreement with the TR^3 spectra recorded in the absence of the sacrificial electron donor (main paper figure 1) and the steady-state spectrum of P10(e$^-$) (figure 2d, main paper). As the Raman data is on resonance with P10(e$^-$), but off resonance with the P10 ground state, the P10(e$^-$) bleaches dominate the spectrum at early time delays, despite the population being relatively small. The negative bands complicate the analysis of the data but do not prevent it. As P10(e$^-$) is formed through exciton dissociation and exciton quenching by the sacrificial electron donor over the first 100 ps after 400 nm excitation we

see an increase in scattering intensity of the P10(e^-) Raman bands and these become positive bands in the difference spectrum at long-time scales.

Table S2 *The excitation energies and oscillator strengths for the first 3 states of P10$_1$, predicted using equation-of-motion coupled-cluster singles and doubles (EOM-CCSD) and DFT in the presence of a polarisable continuum representing water. The optimized B3LYP geometry was used in the EOM-CCSD calculations.*

	State 1			State 2			State 3		
Level of theory	Energy [eV] (nm)	Osc. Strength [arb.]	$<S^2>$	Energy [eV] (nm)	Osc. Strength [arb.]	$<S^2>$	Energy [eV] (nm)	Osc. Strength [arb.]	$<S^2>$
EOM-CCSD/cc-pVDZ	4.5459 (272.74)	0.0250	-	5.0121 (247.37)	0.0014	-	5.0888 (243.64)	0.1297	-
TD-B3LYP/cc-pVDZ	4.1230 (300.71)	0.1083	-	4.4964 (275.74)	0.0413	-	4.7838 (259.17)	0.0276	-
TD-CAM-B3LYP/cc-pVDZ	4.4869 (276.32)	0.1332	-	4.9122 (252.40)	0.0543	-	5.2045 (238.23)	0.0078	-
TD-ωB97XD/cc-pVDZ	4.5042 (275.26)	0.1354	-	4.9239 (251.80)	0.0518	-	5.1978 (238.53)	0.0075	-

Table S3 *The excitation energies and oscillator strengths for the first 3 states of one-electron reduced P10$_1$, P10$_1$(e$^-$), predicted using equation-of-motion coupled-cluster singles and doubles (EOM-CCSD) and TD-DFT in the presence of a polarisable continuum representing water. The optimized B3LYP geometry was used in the EOM-CCSD calculations*

	State 1			State 2			State 3		
Level of theory	Energy [eV] (nm)	Osc. Strength [arb.]	<S^2>	Energy [eV] (nm)	Osc. Strength [arb.]	<S^2>	Energy [eV] (nm)	Osc. Strength [arb.]	<S^2>
EOM-CCSD/cc-pVDZ	1.2929 (958.98)	0.0068		2.0349 (609.29)	0.0540		2.9516 (420.05)	0.1224	
TD-B3LYP/cc-pVDZ	0.9566 (1296.08)	0.0032	0.770	1.6777 (739.00)	0.0453	0.772	2.6520 (467.52)	0.1186	0.828
TD-CAM-B3LYP/cc-pVDZ	1.1789 (1051.68)	0.0059	0.814	1.8367 (675.03)	0.0742	0.832	2.8161 (440.27)	0.1272	0.959
TD-ωB97XD/cc-pVDZ	1.1905 (1041.48)	0.0061	0.808	1.8471 (671.25)	0.0744	0.825	2.8393 (436.68)	0.1365	0.937

Table S4 *The excitation energies and oscillator strengths for the first 3 states of one-electron oxidised P10$_1$, P10$_1$(h$^+$), predicted using equation-of-motion coupled-cluster singles and doubles (EOM-CCSD) and TD-DFT in the presence of a polarisable continuum representing water. The optimized B3LYP geometry was used in the EOM-CCSD calculations*

Level of theory	Energy [eV] (nm)	Osc. Strength [arb.]	<S^2>	Energy [eV] (nm)	Osc. Strength [arb.]	<S^2>	Energy [eV] (nm)	Osc. Strength [arb.]	<S^2>
EOM-CCSD/cc-pVDZ	1.6491 (751.85)	0.0024		1.7646 (702.62)	0.0001		2.3092 (536.92)	0.2102	
TD-B3LYP/cc-pVDZ	1.1185 (1108.53)	0.0018	0.781	1.1688 (1060.78)	0.0005	0.780	1.5664 (791.50)	0.0004	0.772
TD-CAM-B3LYP/cc-pVDZ	1.4386 (861.87)	0.0024	0.843	1.5696 (789.92)	0.0008	0.849	2.0833 (595.14)	0.2408	0.770
TD-ωB97XD/cc-pVDZ	1.4288 (867.76)	0.0027	0.839	1.5594 (795.10)	0.0008	0.845	2.0459 (606.02)	0.2424	0.767

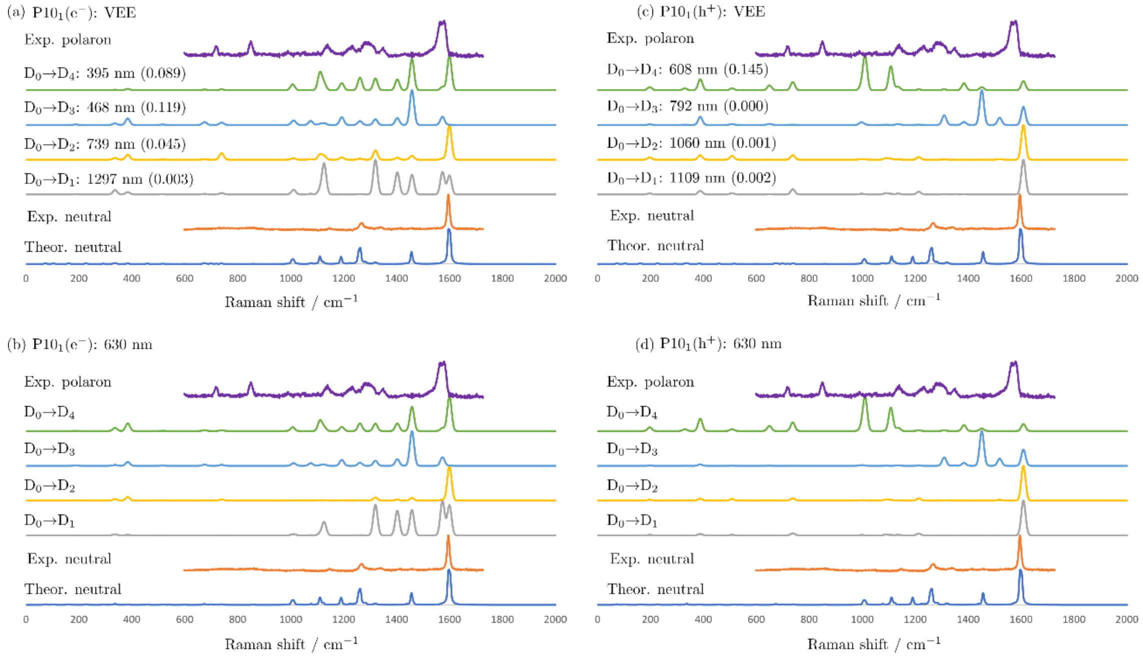

Figure S7. *The solvated resonance Raman spectra of various states of one-electron reduced P10$_1$ where the incident light equals the vertical excitation energy of the specific state (a) or 630 nm (b). The solvated resonance Raman spectra of various states of one-electron oxidized P10$_1$ where the incident light equals the vertical excitation energy of the specific state (c) or 630 nm (d). All spectra were predicted using the ωB97XD exchange-correlation functional and the cc-pVDZ basis set, with a peak half-width at half height of 10* cm^{-1} *and scaled by 0.950. The predicted intensities of each oligomer are normalized to the most intense signal in each spectrum. The predicted excitation energy and oscillator strength is provided for the state and for comparison we include the predicted solvated preresonance Raman spectrum (Theor. neutral) and the experimental spectra of the neutral (Exp. neutral) and polaron (Exp. polaron) materials.*

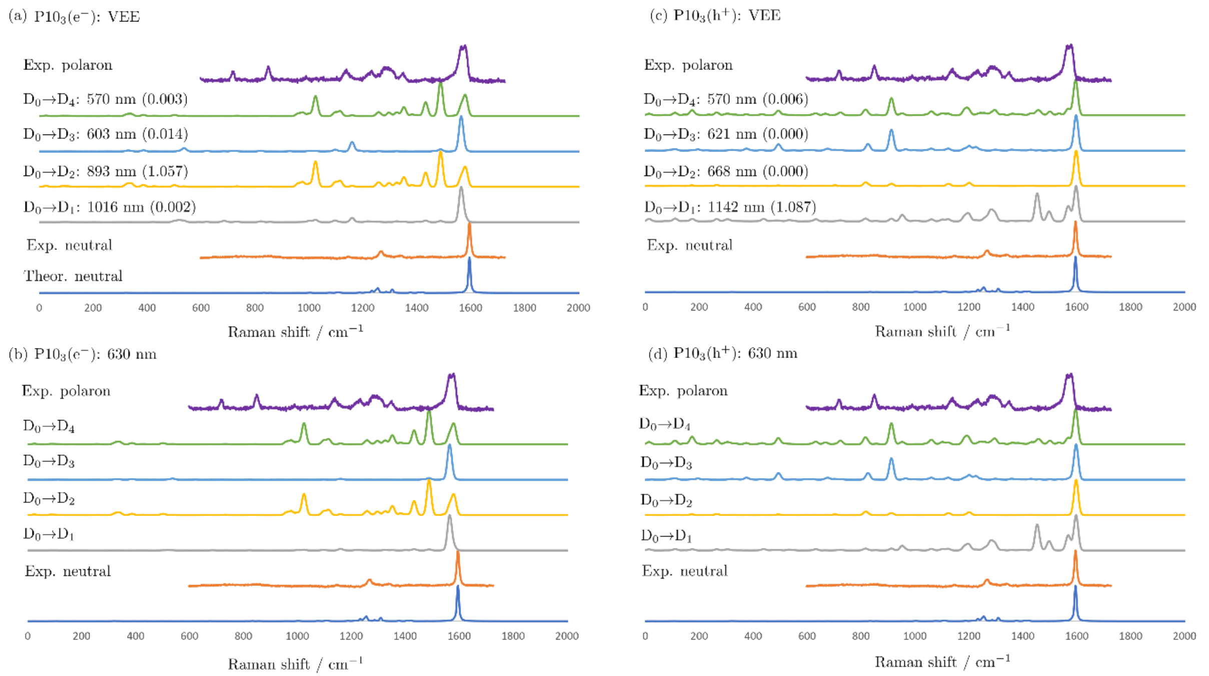

Figure S8. *The solvated resonance Raman spectra of various states of one-electron reduced $P10_3$ where the incident light equals the vertical excitation energy of the specific state (a) or 630 nm (b). The solvated resonance Raman spectra of various states of one-electron oxidized $P10_3$ where the incident light equals the vertical excitation energy of the specific state (c) or 630 nm (d). All spectra were predicted using the ωB97XD exchange-correlation functional and the cc-pVDZ basis set, with a peak half-width at half height of 10* cm^{-1} *and scaled by 0.943. The predicted intensities of each oligomer are normalized to the most intense signal in each spectrum. The predicted excitation energy and oscillator strength is provided for the state and for comparison we include the predicted solvated preresonance Raman spectrum (Theor. neutral) and the experimental spectra of the neutral (Exp. neutral) and polaron (Exp. polaron) materials.*

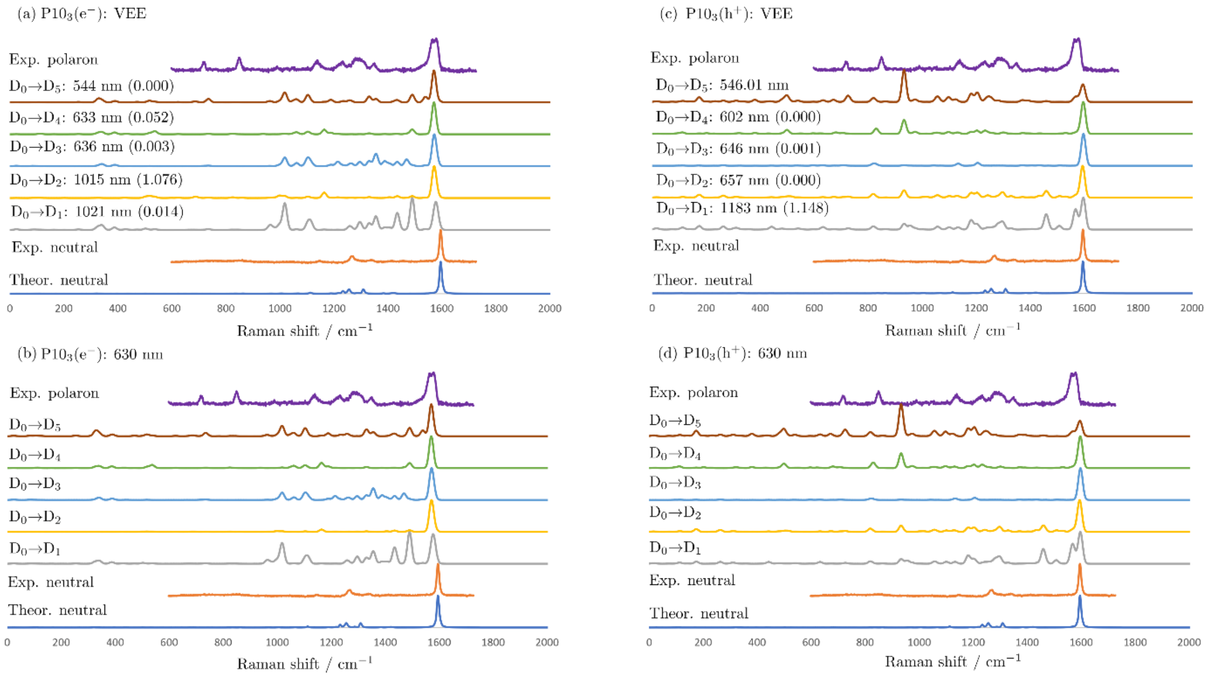

Figure S9. *The solvated resonance Raman spectra of various states of one-electron reduced P10$_3$ where the incident light equals the vertical excitation energy of the specific state (a) or 630 nm (b). The solvated resonance Raman spectra of various states of one-electron oxidized P10$_3$ where the incident light equals the vertical excitation energy of the specific state (c) or 630 nm (d). All spectra were predicted using the CAM-B3LYP exchange-correlation functional and the cc-pVDZ basis set, with a peak half-width at half height of 10* cm^{-1} *and scaled by 0.943. The predicted intensities of each oligomer are normalized to the most intense signal in each spectrum. The predicted excitation energy and oscillator strength is provided for the state and for comparison we include the predicted solvated preresonance Raman spectrum (Theor. neutral) and the experimental spectra of the neutral (Exp. neutral) and polaron (Exp. polaron) materials.*

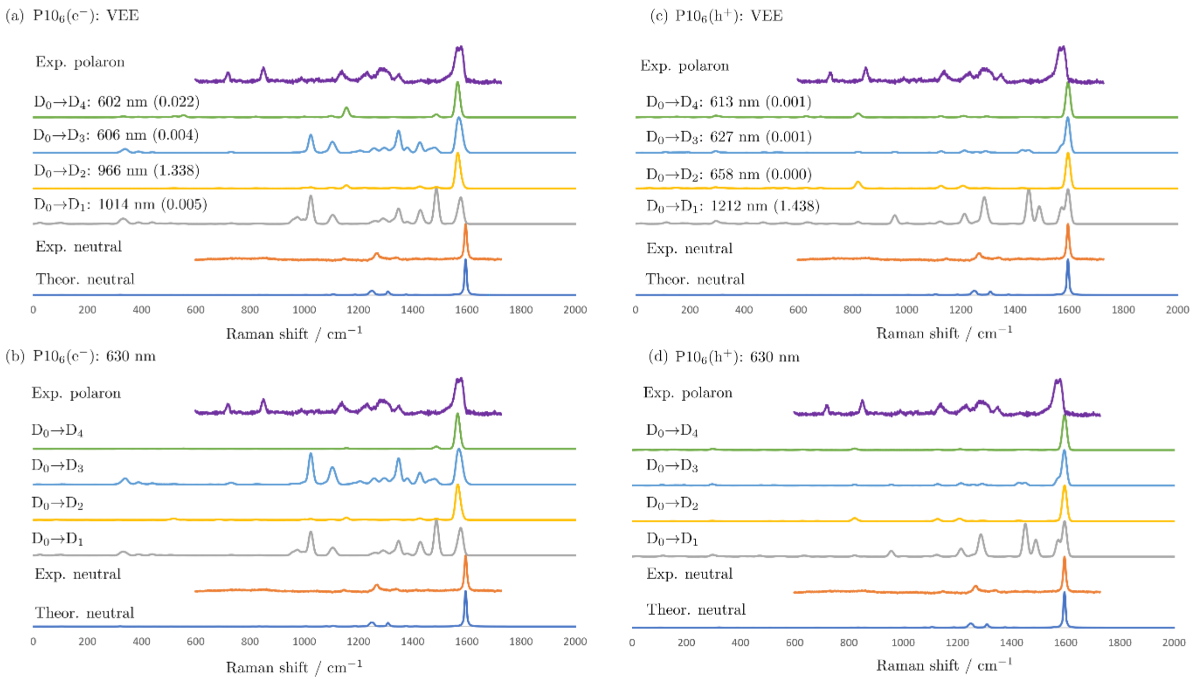

Figure S10. *The solvated resonance Raman spectra of various states of one-electron reduced P10$_6$ where the incident light equals the vertical excitation energy of the specific state (a) or 630 nm (b). The solvated resonance Raman spectra of various states of one-electron oxidized P10$_6$ where the incident light equals the vertical excitation energy of the specific state (c) or 630 nm (d). All spectra were predicted using the ωB97XD exchange-correlation functional and the cc-pVDZ basis set, with a peak half-width at half height of 10* cm^{-1} *and scaled by 0.943. The predicted intensities of each oligomer are normalized to the most intense signal in each spectrum. The predicted excitation energy and oscillator strength is provided for the state and for comparison we include the predicted solvated preresonance Raman spectrum (Theor. neutral) and the experimental spectra of the neutral (Exp. neutral) and polaron (Exp. polaron) materials.*

Table S5 *Fitting parameters for the transient Raman bands discussed in the main text*

Sample	cm^{-1}	A_1	τ_1 (ps)	A_2	τ_2 (ps)	A_3	τ_3 (ps)
P10 powder	847	-1.0	3.2 (± 0.8)	0.76	101 (± 26)		
P10 powder	1265	0.85	1.2 (± 1.5)	0.34	18 (± 10)	0.37	360 (± 85)
P10 (MeOH, TEA, H_2O)	847	-6.4	1.3 (± 1.1)	-0.54	20 (± 11)	0.11	> 2000

The time resolution of the TR^3 experiment is limited due to the pulse duration of the laser system (2 ps) means that τ_1 and A_1 have significant uncertainties associated with them. For P10 in the presence of MeOH/TEA/H_2O (847 cm^{-1}) and for the P10 powder (1265 cm^{-1}) the values of τ_1 is reported as < 2 ps in the main text. The ground state bleach is monitored at 1265 cm^{-1}.

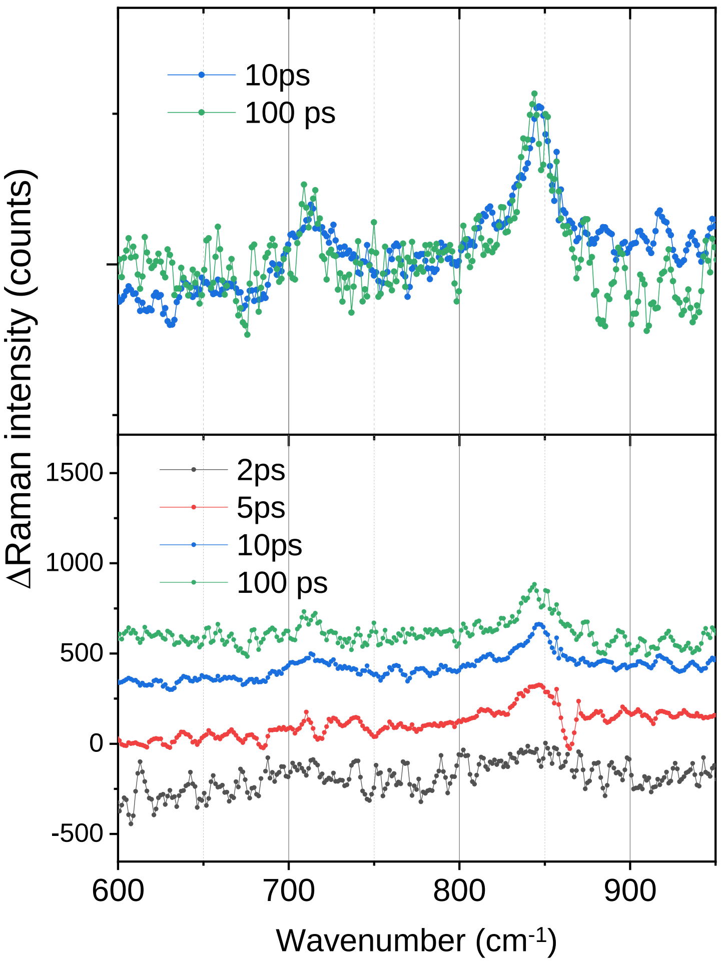

Figure S11. *Expansion of the TR*^{3} *data of P10 powder at the delays indicated (bottom) between 600 and 950 cm*$^{-1}$ *and an overlay of the 10 ps and 100 ps spectra (top). In the 2 and 5 ps spectra the new transient bands are significantly broadened, at times after 10 ps no further change in peak width is observed indicating that cooling is completed within 10 ps.*

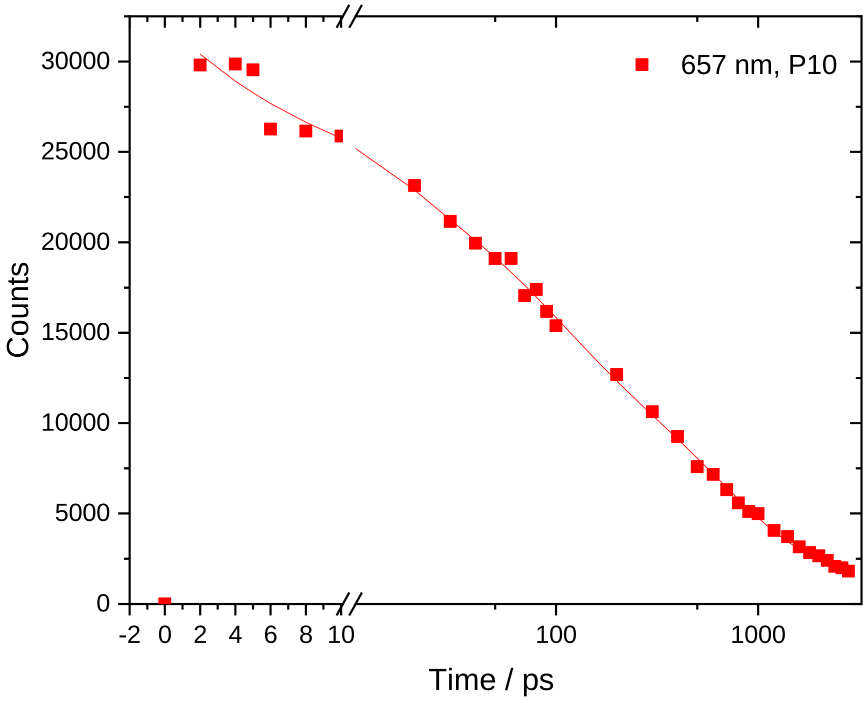

Figure S12. *Time resolved PL data (657 nm) recorded using the Kerr gated spectrometer for P10 powder. The red squares are the recorded signal counts and the line is the result of a fit to 3 exponentials, the minimum required to achieve an adequate fit. See Table S6 for the fitting parameters.*

Table S6 *Fitting parameters for the PL data recorded at 657 nm*

Sample	A_1	τ_1 (ps)	A_2	τ_2 (ps)	A_3	τ_3 (ps)	AWL (ps)
P10 powder	7541	8.9 (± 3.1)	9619	78 (± 25)	13174	662 (± 104)	314
P10 (MeOH, TEA, H_2O)	8717	12.6 (± 0.7)	2748	241 (± 38)			67

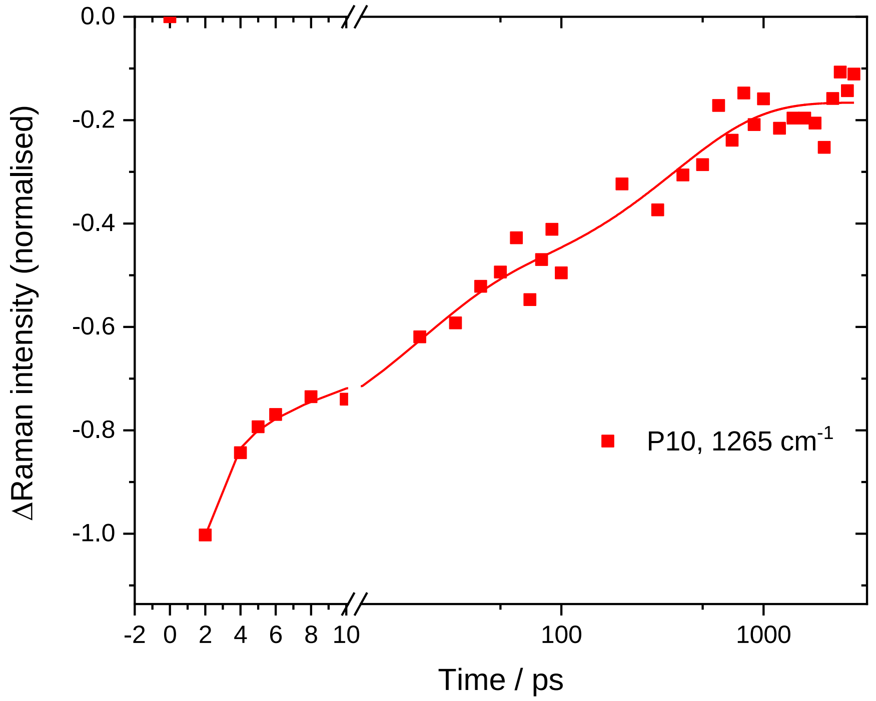

Figure S13. *TR[3] data of the P10 ground state bleach recorded at 1265 cm^{-1}*

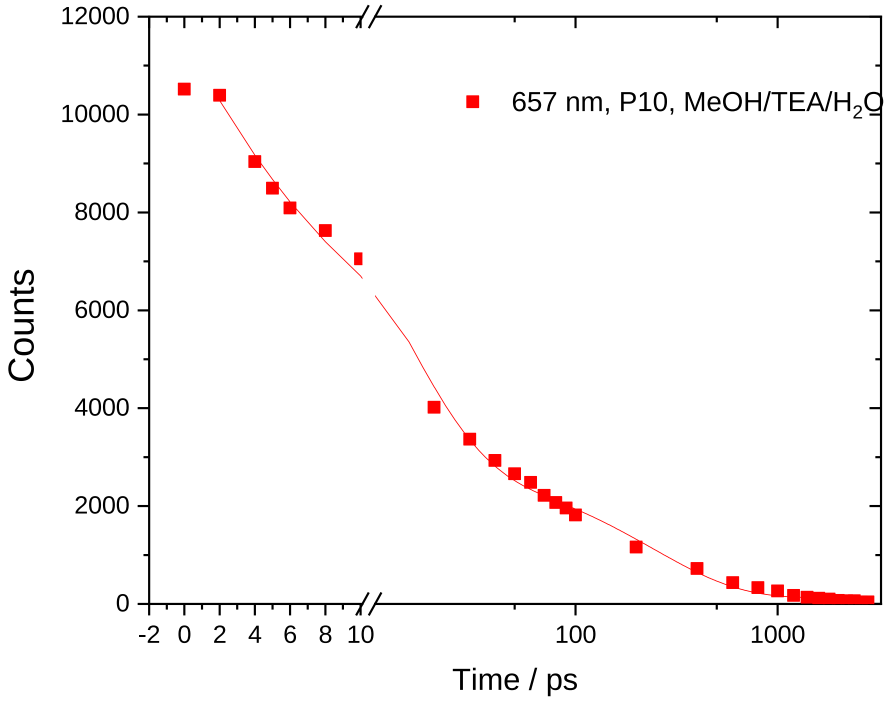

Figure S14. *Time resolved PL data (657 nm) recorded using the Kerr gated spectrometer for P10 in the presence of MeOH/TEA/H_2O. The red squares are the recorded signal counts and the line is the result of a fit to 3 exponentials, the minimum required to achieve an adequate fit. See Table S6 for the fitting parameters.*

3. References

(1) Sachs, M.; Sprick, R. S.; Pearce, D.; Hillman, S. A. J.; Monti, A.; Guilbert, A. A. Y.; Brownbill, N. J.; Dimitrov, S.; Shi, X.; Blanc, F.; Zwijnenburg, M. A.; Nelson, J.; Durrant, J. R.; Cooper, A. I. Understanding Structure-Activity Relationships in Linear Polymer Photocatalysts for Hydrogen Evolution. *Nat. Commun.* **2018**, *9* (1), 4968. https://doi.org/10.1038/s41467-018-07420-6.

(2) Parker, A. W.; Robinson, D. A.; Greetham, G. M.; Clark, I. P.; Pollard, M. R.; Towrie, M.; George, M. W.; Kogimtzis, M.; Matousek, P.; Codd, P. S.; Burgos, P.; Cao, Q.; Farrow, R. C.; Xin, Z.-J. ULTRA: A Unique Instrument for Time-Resolved Spectroscopy. *Appl. Spectrosc. Vol. 64, Issue 12, pp. 1311-1319* **2010**, *64* (12), 1311–1319.

(3) Benniston, A. C.; Matousek, P.; McCulloch, I. E.; Parker, A. W.; Towrie, M. Detailed Picosecond Kerr-Gated Time-Resolved Resonance Raman Spectroscopy and Time-Resolved Emission Studies of Merocyanine 540 in Various Solvents. *J. Phys. Chem. A* **2003**, *107* (22), 4347–4353. https://doi.org/10.1021/JP027343Z.

(4) Keshari, S.; And, S.; Umapathy, S.; Parker, A. W. Focal Point Review Time-Resolved Resonance Raman Spectroscopy: Exploring Reactive Intermediates. *Appl. Spectrosc. Vol. 65, Issue 10, pp. 1087-1115* **2011**, *65* (10), 1087–1115. https://doi.org/10.1366/11-06406.

(5) Stanley, A.; Parker, A. W.; Towrie, M.; Matousek, P. Efficient Rejection of Fluorescence from Raman Spectra Using Picosecond Kerr Gating. *Appl. Spectrosc. Vol. 53, Issue 12, pp. 1485-1489* **1999**, *53* (12), 1485–1489.

(6) Fu, Z.; Wang, X.; Gardner, A. M.; Wang, X.; Chong, S. Y.; Neri, G.; Cowan, A. J.; Liu, L.; Li, X.; Vogel, A.; Clowes, R.; Bilton, M.; Chen, L.; Sprick, R. S.; Cooper, A.

I. A Stable Covalent Organic Framework for Photocatalytic Carbon Dioxide Reduction. *Chem. Sci.* **2020**, *11* (2), 543–550. https://doi.org/10.1039/C9SC03800K.

(7) Hohenberg, P.; Kohn, W. Inhomogeneous Electron Gas. *Phys. Rev.* **1964**, *136* (3B), B864. https://doi.org/10.1103/PhysRev.136.B864.

(8) Kohn, W.; Sham, L. J. Self-Consistent Equations Including Exchange and Correlation Effects. *Phys. Rev.* **1965**, *140* (4A), A1133. https://doi.org/10.1103/PhysRev.140.A1133.

(9) Frisch, M. J.; Trucks, G. W.; Schlegel, H. B.; Scuseria, G. E.; Robb, M. A.; Cheeseman, J. R.; Scalmani, G.; Barone, V.; Petersson, G. A.; Nakatsuji, H.; Li, X.; Caricato, M.; Marenich, A. V.; Bloino, J.; Janesko, B. G.; Gomperts, R.; Mennucci, B.; Hratch, D. J. Gaussian 16 (Revision A.03). Gaussian Inc., Wallingford.

(10) Miertuš, S.; Scrocco, E.; Tomasi, J. Electrostatic Interaction of a Solute with a Continuum. A Direct Utilizaion of AB Initio Molecular Potentials for the Prevision of Solvent Effects. *Chem. Phys.* **1981**, *55* (1), 117–129. https://doi.org/10.1016/0301-0104(81)85090-2.

(11) Cammi, R.; Tomasi, J. Remarks on the Use of the Apparent Surface Charges (ASC) Methods in Solvation Problems: Iterative versus Matrix-Inversion Procedures and the Renormalization of the Apparent Charges. *J. Comput. Chem.* **1995**, *16* (12), 1449–1458. https://doi.org/10.1002/JCC.540161202.

(12) Scalmani, G.; Frisch, M. J. Continuous Surface Charge Polarizable Continuum Models of Solvation. I. General Formalism. *J. Chem. Phys.* **2010**, *132* (11), 114110. https://doi.org/10.1063/1.3359469.

(13) Jacopo Tomasi, *,†; Benedetta Mennucci, † and; Cammi‡, R. Quantum Mechanical

Continuum Solvation Models. *Chem. Rev.* **2005**, *105* (8), 2999–3093. https://doi.org/10.1021/CR9904009.

(14) Cheeseman, J. R.; Frisch, M. J. Basis Set Dependence of Vibrational Raman and Raman Optical Activity Intensities. *J. Chem. Theory Comput.* **2011**, *7* (10), 3323–3334. https://doi.org/10.1021/CT200507E.

(15) Baiardi, A.; Bloino, J.; Barone, V. A General Time-Dependent Route to Resonance-Raman Spectroscopy Including Franck-Condon, Herzberg-Teller and Duschinsky Effects. *J. Chem. Phys.* **2014**, *141* (11). https://doi.org/10.1063/1.4895534.

(16) Becke, A. D. A New Mixing of Hartree–Fock and Local Density-functional Theories. *J. Chem. Phys.* **1998**, *98* (2), 1372. https://doi.org/10.1063/1.464304.

(17) Stephens, P. J.; Devlin, F. J.; Chabalowski, C. F.; Frisch, M. J. Ab Initio Calculation of Vibrational Absorption and Circular Dichroism Spectra Using Density Functional Force Fields. *J. Phys. Chem.* **2002**, *98* (45), 11623–11627. https://doi.org/10.1021/J100096A001.

(18) Lee, C.; Yang, W.; Parr, R. G. Development of the Colle-Salvetti Correlation-Energy Formula into a Functional of the Electron Density. *Phys. Rev. B* **1988**, *37* (2), 785. https://doi.org/10.1103/PhysRevB.37.785.

(19) Yanai, T.; Tew, D. P.; Handy, N. C. A New Hybrid Exchange–Correlation Functional Using the Coulomb-Attenuating Method (CAM-B3LYP). *Chem. Phys. Lett.* **2004**, *393* (1–3), 51–57. https://doi.org/10.1016/J.CPLETT.2004.06.011.

(20) Chai, J.-D.; Head-Gordon, M. Long-Range Corrected Hybrid Density Functionals with Damped Atom–Atom Dispersion Corrections. *Phys. Chem. Chem. Phys.* **2008**, *10* (44), 6615–6620. https://doi.org/10.1039/B810189B.

(21) Grimme, S.; Antony, J.; Ehrlich, S.; Krieg, H. A Consistent and Accurate Ab Initio Parametrization of Density Functional Dispersion Correction (DFT-D) for the 94 Elements H-Pu. *J. Chem. Phys.* **2010**, *132* (15), 154104. https://doi.org/10.1063/1.3382344.

(22) Grimme, S. Semiempirical GGA-Type Density Functional Constructed with a Long-Range Dispersion Correction. *J. Comput. Chem.* **2006**, *27* (15), 1787–1799. https://doi.org/10.1002/JCC.20495.

(23) Runge, E.; Gross, E. K. U. Density-Functional Theory for Time-Dependent Systems. *Phys. Rev. Lett.* **1984**, *52* (12), 997. https://doi.org/10.1103/PhysRevLett.52.997.

(24) III, G. D. P.; Bartlett, R. J. A Full Coupled-cluster Singles and Doubles Model: The Inclusion of Disconnected Triples. *J. Chem. Phys.* **1998**, *76* (4), 1910. https://doi.org/10.1063/1.443164.

(25) Scuseria, G. E.; Janssen, C. L.; III, H. F. S. An Efficient Reformulation of the Closed-shell Coupled Cluster Single and Double Excitation (CCSD) Equations. *J. Chem. Phys.* **1998**, *89* (12), 7382. https://doi.org/10.1063/1.455269.

(26) Scuseria, G. E.; III, H. F. S. Is Coupled Cluster Singles and Doubles (CCSD) More Computationally Intensive than Quadratic Configuration Interaction (QCISD)? *J. Chem. Phys.* **1998**, *90* (7), 3700. https://doi.org/10.1063/1.455827.

(27) Stanton, J. F.; Bartlett, R. J. The Equation of Motion Coupled-cluster Method. A Systematic Biorthogonal Approach to Molecular Excitation Energies, Transition Probabilities, and Excited State Properties. *J. Chem. Phys.* **1998**, *98* (9), 7029. https://doi.org/10.1063/1.464746.

(28) van Stokkum, I. H. M.; Larsen, D. S.; van Grondelle, R. Global and Target Analysis of

Time-Resolved Spectra. *Biochim. Biophys. Acta - Bioenerg.* **2004**, *1657* (2–3), 82–104. https://doi.org/10.1016/j.bbabio.2004.04.011.
